# Power-Tool Use in Orthopaedic Surgery: Iatrogenic Injury, Its Detection, and Technological Advances: A Systematic Review

**DOI:** 10.2106/JBJS.OA.21.00013

**Published:** 2021-11-19

**Authors:** Matthew C.A. Arnold, Sarah Zhao, Ruben J. Doyle, Jonathan R.T. Jeffers, Oliver R. Boughton

**Affiliations:** 1 The MSk Lab, Imperial College London, London, United Kingdom; 2 Department of Mechanical Engineering, Imperial College London, London, United Kingdom

## Abstract

Power tools are an integral part of orthopaedic surgery but have the capacity to cause iatrogenic injury. With this systematic review, we aimed to investigate the prevalence of iatrogenic injury due to the use of power tools in orthopaedic surgery and to discuss the current methods that can be used to reduce injury.

**Methods:**

We performed a systematic review of English-language studies related to power tools and iatrogenic injuries using a keyword search in MEDLINE, Embase, PubMed, and Scopus databases. Exclusion criteria included injuries related to cast-saw use, temperature-induced damage, and complications not clearly related to power-tool use.

**Results:**

A total of 3,694 abstracts were retrieved, and 88 studies were included in the final analysis. Few studies and individual case reports looked directly at the prevalence of injury due to power tools. These included 2 studies looking at the frequency of vascular injury during femoral fracture fixation (0.49% and 0.2%), 2 studies investigating the frequency of vertebral artery injury during spinal surgery (0.5% and 0.08%), and 4 studies investigating vascular injury during total joint arthroplasty (1 study involving 138 vascular injuries in 124 patients, 2 studies noting 0.13% and 0.1% incidence, and 1 questionnaire sent electronically to surgeons). There are multiple methods for preventing damage during power-tool use. These include the use of robotics and simulation, specific drill settings, and real-time feedback techniques such as spectroscopy and electromyography.

**Conclusions:**

Power tools have the potential to cause iatrogenic injury to surrounding structures during orthopaedic surgery. Fortunately, the published literature suggests that the frequency of iatrogenic injury using orthopaedic power tools is low. There are multiple technologies available to reduce damage using power tools. In high-risk operations, the use of advanced technologies to reduce the chance of iatrogenic injury should be considered.

**Level of Evidence:**

Therapeutic Level IV. See Instructions for Authors for a complete description of levels of evidence.

Power-tool use has become integral to orthopaedic surgery by increasing precision and efficiency of bone drilling/sawing in comparison to the use of manual hand tools^1^. These instruments typically consist of a power source within a motorized handpiece, which can be operated at varying speeds and used with a range of burr, saw, blade, drill, and screwdriver tip attachments^2^. Their clinical applications within orthopaedic surgery include enabling deeper access through bone material, debriding tissue, and preparing bone material for orthopaedic implants (such as during fracture fixation, which commonly requires the careful placement of drill holes)^1^. However, despite the wide applications, power-tool use has its disadvantages. These include thermal necrosis^3^, breakage of drill bits^4^, damage to surrounding tissue structures, and overdrilling/oversawing^5,6^. Knowledge of these events would be useful not only to the surgeon operating the device but also to engineers aiming to develop improved and safer medical technology. In other specialties, we have seen demand for, and the development of, increasingly safety-focused tools, for example, tissue-selective piezoelectric drills in head and neck surgery and self-stopping drills in neurosurgery^7,8^. Iatrogenic injuries in orthopaedics include vascular^9^, neurological^10^, and tendon^11^ injuries, and they can have severe consequences; a previous study suggested that 7.3% of vascular injuries in total hip arthroplasty result in death^12^. Several methods, such as robotics^13^, novel drill technology^14^, and real-time feedback mechanisms^15^, are being developed to reduce these injuries.

The aim of this systematic review was to estimate the prevalence of iatrogenic injuries due to power-tool use in patients undergoing orthopaedic surgery and to discuss the current methods to reduce injury and thus improve patient safety.

## Materials and Methods

### Search and Information Sources

The methodology of this review is reported according to the Preferred Reporting Items for Systematic Reviews and Meta-Analyses (PRISMA) guidelines^16^. A systematic review of published literature relating to power tools and iatrogenic injury was undertaken via MEDLINE, Embase, PubMed, and Scopus databases up to April 1, 2020. We used a combination of the search terms “overdrill,” “oversaw,” “tool,” “injury,” “danger,” “safe,” “damage,” “overshot,” “risk,” “drill,” “saw,” “iatrogenic,” “hospital acquired condition,” “medical errors,” “medical mistake,” and “orthopaedic”. The electronic database search was further supplemented by manual review of the references within key relevant studies.

### Eligibility Criteria

Studies were included if they reported on an orthopaedic procedure that featured the use of a power tool. All study types were eligible, including case reports, animal research and simulation studies, cohort studies, and literature reviews. Included studies were either written in, or translated to, the English language.

Studies were excluded if they involved injuries related to cast-saw, arthroscopic trocar, diathermy, needle, scissors, and blade use. Studies relating to complications outside the scope of orthopaedic surgery, injuries not clearly related to intraoperative power-tool use, or injuries related to temperature-induced damage or infection were also excluded.

The 2 outcomes measured were the prevalence of iatrogenic injury related to power-tool use and the methods or safety mechanisms present to reduce iatrogenic injury.

The first 2 authors (M.C.A.A. and S.Z.) independently conducted the search, screened abstracts, and selected studies for review. Any discrepancies regarding article inclusion were resolved via discussion as recommended by the Cochrane Collaboration guidelines^17^.

### Data Collection Process

Relevant data items were extracted from each included article and are presented in Table I. Items included were type of bone, type of iatrogenic injury, power tool used, type of operative procedure, and outcome/recommendation by the authors. The papers were then categorized according to prevalence of iatrogenic injuries, methods to reduce damage using power tools, methods to detect damage when using power tools, and recommendations for power-tool settings.

**TABLE I:** Papers Included in Qualitative Synthesis*

Year	Study	Type of Bone	Type of Study	Type of Injury	Tool Use	Operative Procedure	Recommendation or Outcome
2012	Alajmo et al.^5^	Artificial bone	Laboratory	NA	Synthes	Drilling	Blunt drill bits significantly worsen plunging compared with sharp drill bits
2019	Alam et al.^75^	Bovine femur	Laboratory	Bone stress/necrosis/cracks, drill breakage	Vibrational drilling	Drilling	The drilling force, torque, temperature, and cell loss could be minimized when the drill speed was maintained at 1,000 rpm, the feed rate at 30 mm/min, and the frequency at 20 kHz
2015	Alshameeri et al.^12^	Hip joint	Systematic review	Vascular	NA	Total hip arthroplasty	Femoral and external iliac arteries have the highest incidence of vascular injury
2012	Aziz et al.^67^	Bovine femur	Laboratory	NA	CRS Catalyst-5 robot (Thermo CRS)	Drilling	The algorithm can be used to prevent any drill-bit breakage, unnecessary drill-bit insertion, and any mechanical damage to the bone
2010	Bail et al.^56^	Talus	Cadaveric	NA	3.4-mm titanium spiral drill bit	Drilling of osteochondral lesions	MRI appears to be a viable imaging technique
2015	Barquet et al.^6^	Femur	Systematic review	Vascular	NA	Internal fixation (varying types)	Incidence of morbidity and mortality of vascular injury from femoral fracture fixation was 11.44% and 6.62%, respectively
2013	Boiadjiev et al.^35^	Bovine femur	Laboratory	NA	Passive navigation principle for orthopaedic interventions with MR fluoroscopy	Nonspecified drilling	Robotic use in surgery is still under development but could be a useful tool in the future
2006	Bolger et al.^61^	Porcine spine	Laboratory	Pedicle breach	Custom-made device	Pedicle drilling	Use of impedance measurement with drill tool allows real-time detection of pedicle perforation
2007	Bolger et al.^62^	Spine	Clinical trial	Pedicle breach	PediGuard (SpineVision)	Pedicle-screw fixation	Electrical conductivity measurement may provide a simple, safe, and sensitive method of detecting pedicle breaches
2019	Butler and Halter^65^	Swine femur	Laboratory	Not specified	OsseoSet 200 system (Nobel Biocare)	Drilling	The system can prevent iatrogenic injury associated with breaching the inferior alveolar nerve or maxillary sinus
2010	Butt et al.^77^	Knee joint	Review	Vascular	NA	Total knee arthroplasty	There are 4 mechanisms for arterial injury during total knee arthroplasty, 1 of which includes direct injury to the vessel with power tools
2013	Bydon et al.^43^	Spine	Retrospective case series	Durotomy	Ultrasonic bone curette and high-speed drill	Spinal decompression	The ultrasonic bone curette has a safety profile similar to that of the high-speed drill
2003	Calligaro et al.^21^	Hip and knee joint	Retrospective case series	Vascular	NA	Hip and knee arthroplasty	There were acute arterial complications in 32 patients (0.13%)
2010	Cartiaux et al.^34^	Artificial bone	Laboratory	NA	Compact Air Drive II (Synthes)	NA	There was a significant increase in accuracy when a robotic device was used in conjunction with an oscillating saw
2012	Clement et al.^37^	Artificial bone	Laboratory	NA	Small fragment drill and air drill (Synthes)	NA	Experienced surgeons penetrated the far cortex by a mean of 6.33 mm
2003	Da Silva et al.^22^	Knee joint	Survey	Popliteal artery	NA	Total knee arthroplasty	Popliteal artery injury during total knee arthroplasty is primarily the result of direct trauma to the vessel
2014	Dai et al.^76^	Porcine spine	Laboratory	NA	Laser displacement sensor measuring vibration	Drilling	Minimizes radiation exposure and allows real-time feedback
2016	den Dunnen et al.^78^	Porcine talus and femur	Laboratory	NA	Custom-made water jet	Drilling	The most accurate results in drilling depth can be achieved by applying a nozzle of 0.4 mm, a pressure of 50 MPa, and jet times between 1 and 5 s.
2019	Di Martino et al.^64^	Cervical spine	Systematic review	Neurovascular injury	NA	Cervical spine decompression	Evoked-potential monitoring has a high sensitivity and specificity for detecting neural damage, but it is unclear which patients it is indicated for
2019	Duan et al.^30^	Femur	Prospective case series	NA	TiRobot system (TINAVI Medical Technologies)	Percutaneous cannulated screw fixation	Robot-assisted screw fixation allows accurate screw insertion, less invasion, and less radiation exposure
2019	Duperron et al.^15^	Bovine femur	Laboratory	NA	Modified version of surgical hand-drill (CD4; Stryker U.S.A.)	Intramedullary nailing	Diffuse reflectance spectroscopy can be successfully integrated into a handheld drill
1992	Elliott^1^	Tibia	Laboratory	NA	AO Small Air Drill (Straumann U.K.)	Dynamic hip screw	Drills do not increase risk of damage and increase operative time by 30 s per screw
2011	Flannery et al.^50^	Tibia	Cadaveric	Plunging and nerve overwrapping	Handheld cordless drill (Standard Stryker drill)	Screw placement	Structures are at higher risk when using a threaded pin versus a smooth pin
2018	Franzini et al.^44^	Spine	Case series	Spinal root, dura mater, venous plexus spinal cord injury	Mectron piezosurgery device (Mectron Medical Technology)	Laminoplasty	Piezoelectric device has good safety and precision profile
2018	Gilmer and Lang^14^	Artificial bone	Laboratory	Fracture	Custom made dual motor drill	Screw placement	Measurement of drilling energy allowed for calculation of bone density, which correlated very strongly with the known density
2010	Gras et al.^55^	Pelvis	Radiographic	Prevesical hematoma	2D fluoroscopic navigation	Screw placement	Provides high accuracy of screw placement, but for bilateral iliosacral screw fixation, 3D fluoroscopy is preferred
2011	Gras et al.^58^	Knee and talus	Case series	NA	Optoelectronic system for navigation of the drill and target reference pointer	Drilling	Increase drilling precision and reduce radiation exposure by reducing use of fluoroscopy
2004	Grauer et al.^79^	Spine	Cadaveric	Neurological injury	SafePath cannulation device	Pedicle-screw insertion	May be better for pedicle-screw insertion cannulation in lumbar spine compared with standard techniques
2019	Hampp et al.^13^	Knee	Cadaveric	Soft-tissue injury	The RATKA (robotic arm-assisted total knee arthroplasty) system (Mako Surgical Corp. [Stryker])	Total knee arthroplasty	Robotic-assisted total knee arthroplasty may reduce soft-tissue injury, particularly the posterior cruciate ligament
2020	Herregodts et al.^72^	Knee	Cadaveric	Soft-tissue injury	Dyonics power oscillating saw (Smith & Nephew)	Total knee arthroplasty	The oscillating saw significantly passes the edge of the bone during tibial resection in total knee arthroplasty
2019	Itoh et al.^24^	Tibia	Case report/cadaveric study	Deep peroneal nerve injury	Not specified	Medial open‐wedge high tibial osteotomy	Deep peroneal nerve has a risk of injury during distal locking-screw placement in this procedure
1993	Jackson et al.^25^	Femur	Case report	Popliteal artery, tibial/common peroneal nerves	Not specified	Posterior cruciate ligament reconstruction	Square-shouldered drill bit causes higher risk of neurovascular injury as guide pins are used in more distal anatomical insertion
2013	James et al.^80^	Bovine femur	Laboratory	NA	Saw blades (KM-458, Brasseler U.S.A.)	Sawing	Thrust force will always be greater than cutting force for the range of velocities and depths of cut investigated
2016	Jiang et al.^81^	Spine	Case series	Spinal canal entry	Not specified	Atlantoaxial pedicle-screw fixation	Use of novel drill guide template for atlantoaxial pedicle-screw placement is feasible and has high accuracy
2000	Jingushi et al.^82^	Femur	Case series	Perforation and femoral fracture	High-powered drill with variable-sized metal donut attached 3 cm proximal to drill tip end	Removal of femoral cement	Use of high-powered drill equipped with centralizer to remove the distal cement during hip revision arthroplasty can lessen the incidence of femoral perforation
2017	Kamara et al.^83^	Ilium, femur, tibia	Retrospective cohort study	Infection, neurapraxia, suture abscess	Not specified	Hip and knee arthroplasty	Pins required for navigation-assisted arthroplasty have a low complication rate; however, transcortical/juxtacortical drilling is a possible risk factor for pin-site infection
2019	Kazum et al.^38^	Synthetic femur model	Prospective observational study	NA	Power drill, 2.7-mm drill bit (Synthes)	Drilling	Training surgeons on a reproducible and reliable drilling simulator can reduce plunging distance
2009	Khokhotva et al.^84^	Lamb femur	Laboratory	Plunging	Nitrogen-powered surgical drill AO Drill Reamer; Hall Series 4, Model 5067 (Zimmer)	Drilling	Feedback related to plunging does not improve results
2016	Kim et al.^26^	Tibia	Case report	Anterior tibial artery	Not specified	Anterior cruciate ligament (ACL) reconstruction	Drilling for tibial bicortical fixation during ACL reconstruction can directly injure the anterior tibial artery
2016	Kim et al.^85^	Femur	Laboratory	Screw malposition	Antegrade Femoral Nail (Synthes)	Intramedullary nailing	Targeting-device malalignment can occur when placing the proximal reconstruction screws in a reconstruction nailing system
2003	König et al.^45^	Ilium, femur, tibia	Case series	NA	Piezoelectric MRI drilling machine (MRI Devices Daum)	Transcortical bone biopsy	Piezoelectric drill is a safe method for transcortical bone biopsy
2003	Kotani et al.^59^	Spine	Case series	Pedicle wall/anterior vertebral-body-wall perforation	Not specified	Screw insertion	Computer-navigation system can reduce complications related to pedicle-screw insertion
2012	Larson et al.^60^	Spine	Retrospective study	NA	Not specified	Screw insertion	Navigation increases accuracy for spinal instrumentation in congenital spine deformity
2019	Lee et al.^19^	Cervical spine	Retrospective case series	Vertebral artery injury	Not specified	Cervical spine surgery	Overall incidence of vertebral artery injury was 0.08%. C1-2 posterior fixation had the highest incidence (1.35%)
2019	Liebmann et al.^86^	Artificial bone	Laboratory	Neurovascular injury	NA	Pedicle-screw placement	Precise pedicle-screw insertion can be achieved using this method on synthetic bone
2017	Mahylis et al.^11^	Upper limb	Cadaveric	Extensor tendon injury	Continuous or oscillating drill modes	Drilling	Complete extensor tendon failure due to drill-penetration injury is rare
2019	Massimi et al.^46^	Cranium	Retrospective case series	Dural tears	Piezosurgery (Mectron)	Craniotomy/laminotomy	Piezosurgery is a safe and effective alternative to traditional drilling systems
1998	Moed et al.^63^	Pelvis	Case series	Neural injury	2.8-mm drill bit (Synthes) and a 3.2-mm drill bit (Howmedica)	Iliosacral screw fixation of pelvic ring fractures	Electromyography has the potential to reduce neural injury during placement of iliosacral screws
2019	Naik et al.^47^	Orbit	Case series	Infraorbital nerves and vessels	Synthes Piezoelectric System	Orbital floor decompression	Significantly lower chances of infraorbital nerve hypoesthesia when piezoelectric surgery was used
2019	Nam et al.^87^	Spine	Case report	Sacroiliac joint syndrome	Sextant system (Medtronic)	Pedicle-screw insertion	When using the Sextant system, surgeons must be aware of iatrogenic sacroiliac joint syndrome
2019	Neubauer et al.^88^	Femur	Cadaveric	Deep/superficial femoral artery	4-hole DHS system (DePuy Synthes)	Dynamic hip-screw insertion	Deep femoral artery is more at risk than superficial femoral artery with insertion of dynamic hip screw
2013	Pandey and Panda^89^	Multiple	Systematic review	Many	Many	Review	Guidelines for bone drilling include high-speed drill with larger force, supply of coolant, high drill rake angle, use of split point, quick helix, 2-phase drill bit, and large point angle
2014	Pandey and Panda^68^	Bovine femur	Laboratory	NA	MTAB Flexmill	Drilling	The best combination of bone drilling parameters for minimum thrust force is 30 mm/min of feed rate and 1,805 rpm of spindle speed
2008	Parvizi et al.^23^	Hip and knee joints	Retrospective case series	Vascular	NA	Total knee and hip arthroplasty	0.1% (n = 16) of patients were found to have a vascular injury after total hip/knee arthroplasty, with 6 cases attributed to direct arterial injury
2010	Podnar^90^	Spine	Retrospective case series	Cauda equina damage	NA	Lumbar spinal surgery	Lumbar spinal surgery causes a low number of lesions to the cauda equina
2001	Prabhu et al.^27^	Spine	Case report	Vertebral artery pseudoaneurysm	Not specified	Screw fixation	Techniques include immediate removal of the drill, packing with hemostatic agents, angiography, early anticoagulation, and coil embolization with parent vessel occlusion 4 weeks after injury
2020	Puangmali et al.^91^	Nonspecific porcine bone	Laboratory	Not specified	Novel drill device	Drilling	This technique can prevent overdrilling and reduce tissue damage
2010	Qin et al.^92^	Clavicle	Radiographic	Neurovascular bundle damage	NA	Drilling	This study suggests safe zone and optimal drilling depths/angles during internal fixation of clavicular fractures
2017	Ruder et al.^39^	Generic synthetic bone model	Laboratory	Plunging	Not specified	Drilling	There is a reduction in plunging depth with use of a low-cost training model
2004	Safar et al.^28^	Lower limb	Case series	Pseudoaneurysm of axillary artery, popliteal and anterior tibial artery injury, posterior tibial nerve injury, popliteal artery, popliteal vein, tibial nerve	Not specified	Orthopaedic screw placement	This study suggests that the patient should immediately be referred to a vascular surgeon if a high index of suspicion for arterial injury
2014	Schatlo et al.^32^	Spine	Retrospective case series	Neurological injury	SpineAssist (Mazor)	Pedicle screw insertion	Robotic pedicle-screw placement is safe, but there are technical difficulties and, hence, fluoroscopy should also be used
2010	Seebauer et al.^57^	Femur	Cadaveric	NA	MRI-compatible drill and a 3.4-mm titanium drill bit (Invivo)	Osteochondral defect repairs	MRI-assisted navigation method with a passive-navigation device is potentially applicable in the treatment of osteochondral lesions of the knee
2017	Segal et al.^18^	Femur	Retrospective case series	Vascular	Multiple	Internal fixation of intertrochanteric fracture (intermedullary nail or dynamic hip screw)	The rate of iatrogenic vascular injury occurring in internal fixation of intertrochanteric femoral fractures was 0.2% in this study
2019	Shepard et al.^93^	Spine	Cadaveric	Pedicle injury	IntelliSense drill (McGinley Orthopedics)	Pedicle screw insertion	This computerized drill is comparable with a freehand technique at a junior and senior level
2018	Shim et al.^36^	Swine femur	Laboratory	NA	Custom-made robotic system	Nonspecific drilling	The rolling friction mechanism allows immediate drill-tip detachment and enables the robot to have a compact structure
2013	Shin et al.^29^	Pelvis	Case report	Ureter injury	Not specified	Internal fixation of multiple pelvic fractures	When operating on the pelvis, it is important to understand anatomy of the ureter and surrounding structures
2018	Shu et al.^48^	Bovine femur	Laboratory	Bone necrosis	Novel elliptical vibration-assisted orthopaedic oscillating saw	Osteotomy	Elliptical vibration saw may reduce cutting forces during sawing
2017	Singh et al.^53^	Bovine femur	Laboratory	NA	A twist drill	Nonspecific drilling	Lower rotational speed and low feed rate with twist drill provides optimum force and surface roughness
2016	Singh et al.^54^	Bovine femur	Laboratory	NA	CNC vertical milling machine	Nonspecific drilling	Optimal result can be achieved with lower rotational speed (1,000 rpm) and low feed rate (50 mm/min) with twist drill
1993	Smith et al.^20^	Spine	Retrospective case series	Vertebral artery injury	Air drill responsible for most injuries	Vertebral body resection	Provided recommended operative procedure technique
2014	Soriano et al.^51^	Bovine femur	Laboratory	Thermal damage	Multiple	Drilling	A drill bit with 18° rake angle and 0.1-mm margin width reduced temperatures by 50% as well as feed forces and cutting torque by 60% and 50%, respectively
2017	Staats et al.^69^	Multiple sites	Retrospective case series	Nil	Not specified	Tumor resection	Computer-navigated surgery offers a safe tool for resection of musculoskeletal tumors
2017	Stillwell et al.^94^	Clavicle	Cadaveric	Injury to brachial plexus, pseudoaneurysm, arteriovenous fistula, subclavian vein injury	Standard Stryker drill	Plunge depths	Plunging depths were greater for inexperienced surgeons. Medial clavicle most at risk for damage to neurovascular structures
2017	Stranix et al.^40^	Pelvis	Radiographic	Iliac crest bone graft harvest	Simulated drilling	Pelvic visceral injury	Acumed drill-assisted iliac crest bone-graft harvest is a safe technique for obtaining cancellous bone
2019	Sui and Sugita^95^	Bovine femur	Laboratory	Not specified	OKK VM4-2 Machining Center	Drilling	Drilling forces are affected by bone type
2018	Sui and Sugita^52^	Bovine femur	Laboratory	Not specified	OKK VM4-2 Machining Center	Drilling	Optimized drill bits can reduce drilling forces and temperature rise
2016	Synek et al.^96^	Frozen cadaveric radius	Laboratory	Extensor tendon irritation	Not specified	Drilling/screw placement	Self-drilling locking screws can help eliminate overdrilling and distal screw protrusion during fixation of distal radial fractures
2009	Tonetti et al.^41^	Model spine	Laboratory	Not specified	Simulator	Simulation: percutaneous sacroiliac joint screw fixation	Useful simulation for familiarizing surgeons with 2D fluoroscopic guidance in a 3D operating environment
2020	Torun and Pazarci^66^	Artificial bone	Laboratory	Not specified	TRMAX-RTM134 with a MAIER HSS 3.5-mm-diameter and 70-mm-length drill bit	Drilling	This technique could be integrated with the use of conventional drills with minimum configuration changes and allow increased safety when drilling
2017	Tsai et al.^97^	Femur	Cadaveric	Subtrochanteric femoral fracture	Not specified	Drilling	Drilling inferior to the lesser trochanter does not cause an increased chance of fracture compared with drilling at the level of lesser trochanter
2017	Tsai et al.^33^	Spine	Retrospective case series	Not specified	Renaissance Robot-Guided System (Mazor Robotics)	Transpedicle screw placement	Preoperative planning, mounting, registration, execution, and robot assembly may affect the accuracy of pedicle screw placement
2010	Vankipuram et al.^42^	Virtual bone	Laboratory	Not specified	Synthes surgical drill	Drilling	Realistic basic training simulator – visohaptic interaction provides feedback on surgical proficiency
2012	Voormolen et al.^98^	Synthetic material	Laboratory	Temporal bone critical structures	Stealth Treon navigation machine (Medtronic)	Temporal bone drilling	Intraoperative feedback reduces risks of damage to important structures compared with using a standard neuronavigation interface
2019	Wallace et al.^49^	Artificial bone	Laboratory	NA	DeWALT DW1908B	Drilling	Dual motor drill significantly decreases plunge depth regardless of the user’s level of experience
2013	Wetzel et al.^74^	Knee	Cadaveric	Not specified	Oscillating hip vs. tip saw blade	Total knee arthroplasty	No significant difference between oscillating hip and tip saw blades
2019	Wu et al.^31^	NA	Guidance article	NA	TiRobot system for orthopaedic surgery (TINAVI Medical Technologies)	Femoral neck fracture	Robot-assisted orthopaedic surgery provides a less invasive treatment method and reduces radiation exposure
2008	Yau and Chiu^99^	Tibia and femur	Retrospective case series	NA	Passive optical imageless computer navigation system (Brainlab)	Total knee arthroplasty	The average error in the sagittal plane was higher than that in the coronal plane
2013	Yang et al.^100^	Femur	Case report	Femoral fracture	Not specified	Intramedullary nailing	Fluoroscopic imaging should be used to check the fracture line before converting to reconstructive nailing

* NA = not applicable.

### Statistical Analysis

Statistical analysis was not possible because of the heterogeneity of the study types and clinical data mostly coming from case series. Therefore, descriptive statistics were used where possible.

### Source of Funding

This study was supported by a grant from the Wellcome Trust (208858/Z/17/Z). General laboratory funding was provided by the U.K. National Institute for Health Research, The Royal College of Surgeons of England, the Dunhill Medical Trust, and the Michael Uren Foundation.

## Results

**Fig. 1:**
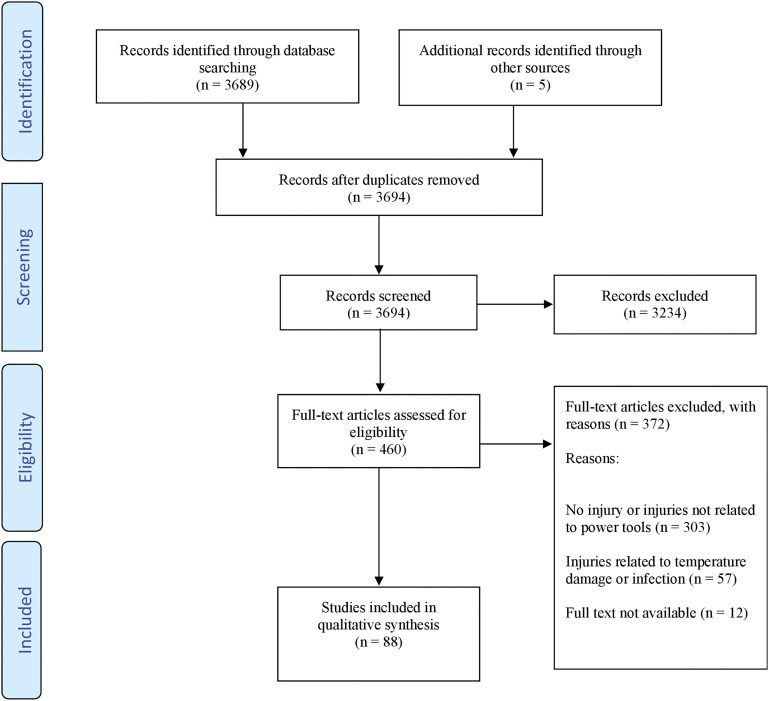
A flow diagram showing the article selection process.

A total of 3,689 abstracts were retrieved via the electronic databases using the search criteria. Five abstracts were included from the manual reference search of relevant papers. Following duplicate removal and abstract screening, a total of 460 eligible full-text studies remained. After review of these full manuscripts, 88 papers were deemed to fit the inclusion criteria and were subsequently included in the systematic review. Figure 1 shows the study selection flow, according to the PRISMA guidelines^16^. Table I lists data collected from all selected studies, with relevant findings and recommendations.

### Prevalence of Iatrogenic Injury Using Power Tools

There were few studies exploring the prevalence of iatrogenic injuries due to power tools. The majority looked at vascular injury, including 2 studies involving femoral fracture fixation^6,18^, 2 involving spinal surgery^19,20^, and 4 involving total joint arthroplasty^12,21-23^. Two of these were systematic reviews that investigated proximal femoral fractures^6^ and total hip arthroplasty^12^, with the majority being retrospective studies and 1 questionnaire sent to vascular surgeons^22^. In addition, there were multiple case reports of arterial, nervous, and ureteric injury due to screw placement and drill bit use^24-29^.

### Novel Methods to Reduce Damage When Using Orthopaedic Power Tools

Nine papers involved robotic systems for use during knee arthroplasty^13^, femoral neck fractures^30,31^, and spinal surgery^32,33^ or in conjunction with sawing^34^ and in laboratory studies^35,36,67^.

Six papers studied simulation^37-42^, with the different types summarized effectively by Vanikipuram et al.^42^. These studies showed that simulation can successfully be used to reduce plunging depth in trainee surgeons^38,39^ and that computer-based simulation was found to provide effective and transferable skills for inexperienced surgeons^40,41^.

Six papers investigated piezoelectric/vibrational drilling^43-48^, 2 studies looked at dual motor drilling^14,49^, and 4 papers were on recommendations for drill bits^5,50-52^. Two studies by the same authors investigated the Taguchi method and suggested a lower rotational speed of 1,000 rpm and feed rate of 50 mm/min with a twist drill to reduce surface roughness and improve drill-hole quality^53,54^.

Six papers looked at imaging techniques in orthopaedic surgery. These include fluoroscopy^55^, magnetic resonance imaging (MRI)^56,57^, fluoro-free navigation techniques^58^, computer-assisted navigation, and 3D image-guided placement^59,60^.

### Methods for Detecting Damage When Using Orthopaedic Power Tools

Numerous papers investigated novel technology to detect damage during orthopaedic surgery. This includes spectroscopy^15^, electrical conductivity devices^61,62^, electromyography^63^, stimulus-evoked potential^64^, bioimpedance drills^65^, and acoustic emission-signal analysis^66^.

### Recommended Settings for Power-Tool Use

Aziz et al. developed an algorithm that detects excessive force and breakthrough of the drill bit during bone drilling, whereby the drill will halt and return to a safe position once the algorithm is triggered^67^. Pandey and Panda calculated the point at which a drill had broken through bone. They found that the best combination of bone-drilling parameters for minimum thrust force is 30 mm/min of feed rate and 1,805 rpm of spindle speed^68^.

## Discussion

This systematic review aimed to determine the prevalence of iatrogenic orthopaedic injuries related to intraoperative power-tool use in the literature and current methods available for reducing the occurrence of these injuries. A total of 88 studies were retrieved and analyzed to help answer these questions, although a wide range of orthopaedic procedures were included. Where possible, we have given recommendations to reduce injury on the basis of the studies. However, our intention was to gain appreciation of the breadth of reported iatrogenic injuries in the literature; providing specific recommendations for each procedure is outside the scope of this review.

### Prevalence of Iatrogenic Injury

Overall, there were few papers that specifically explored the prevalence of iatrogenic power-tool injuries in orthopaedic surgery. Where reported, types of iatrogenic injuries included vascular, nervous, and ureteral injury.

One systematic review from 2015 looked at vascular injuries that occurred during internal fixation of proximal femoral fractures^6^. The authors estimated the incidence of these injuries to be 0.49%. They showed that, of 182 cases of injury identified, 175 were reported as iatrogenic injuries, mostly in the extra-pelvic vessels and specifically the profunda femoris artery. Interestingly, from their analysis, at least 28 of these cases had a confirmed mechanism of injury involving a drill bit. The authors make several recommendations related to power-tool use during internal fixation of proximal femoral fractures. These include encouraging the use of powered instruments under image-intensifier guidance, maintaining the leg in neutral with reduced traction, and keeping the drill bit sharp^6^.

Another systematic review from 2015 investigated the incidence of vascular injury during total hip arthroplasty^12^. The authors described 138 vascular injuries in 124 patients, mostly affecting the common femoral artery (23%) and with the most prevalent mechanism being laceration. However, there was no association between the type of blood vessel injured and surgical approach. The main contributing factors appeared to be aggressive medial retractor placement and injury from screw fixation of the acetabular component. Although not explored in depth during this review, it is important to recognize that the surgeon’s (and assistant’s) knowledge of anatomy and correct retractor placement is vital to reducing the chance of iatrogenic injury. Other retrospective studies looking at arterial injury in total hip/knee arthroplasty found an incidence of 0.13%^21^ and 0.1%^23^, both noting direct laceration as a cause. In addition, a survey sent to vascular surgeons in the U.S. demonstrated 19 instances of popliteal artery injury during total knee arthroplasty (12 cases of which were due to direct injury). However, the response rate was low, with only 13 replies from 190 survey recipients, so underreporting is extremely likely^22^.

Smith et al. conducted a retrospective review of 10 cervical decompression procedures performed by 9 spinal surgeons^20^. They found that the incidence of iatrogenic injury to the vertebral artery was 0.5%, with all cases related to intraoperative motorized power-tool instrumentation. Four of these patients also suffered postoperative neurological deficit, which occurred as a direct result of the arterial injury. The authors give recommendations for avoiding injury, such as dissecting the bone/disc material as close to the midline as possible or using imaging to determine vertebral artery position and artery proximity to the lesion^20^.

A retrospective multicenter study looking at iatrogenic injury to the vertebral artery demonstrated an overall incidence of 0.08%, with C1-2 posterior fixation having the highest incidence (1.35%). This study involved 15,582 surgeries in 21 centers, and 77% of the cases showed no permanent neurological deficit^19^.

### Novel Methods for Reducing Damage When Using Orthopaedic Power Tools

The range of robotic systems in surgery is increasing, with numerous systems developed in the last decade to overcome the inaccuracy of manually navigating orthopaedic tools^35,69^. The benefits of robotic systems include increased safety and a reduced rate of iatrogenic injuries^13,70,71^.

Oscillating saws have the potential to cause soft-tissue damage during total knee arthroplasty^72^, and Cartiaux et al. showed that using robotic navigation in conjunction with these tools has the potential to significantly decrease iatrogenic injury compared with freehand techniques^34^.

Another study looked at robotic-assisted cervical transpedicular screw placement, finding that it achieved 98.8% accuracy in Kirschner wire placement and improved functional outcomes compared with non-robotic-guided placement^33^. Another study of robotic-assisted pedicle screw placement also found increased accuracy in spinal surgery when compared with fluoroscopy-guided techniques^32^.

Shim et al. tested a compact robotic drill prototype using an automated “rolling friction mechanism,” which allowed safe removal of the drill tip in an emergency while not compromising the speed and accuracy of the drill^36^.

Piezoelectric surgery uses high-frequency ultrasonic vibrations to cut bone tissue^73^. When compared with conventional drilling, vibrational drilling aims to reduce force, torque, and thermal damage to bone. This is thought to be possible because of the increased precision and reduced bleeding due to a “microcoagulation” effect^44^. For instance, it has been demonstrated that an elliptical vibration-assisted oscillating saw can minimize required cutting force^48^ and also reduce risk of soft-tissue injury^74^. This technique can be applied safely in a low-field MRI environment and is a valuable method to facilitate transcortical bone biopsy^45^, but there is minimal comparison of this and traditional methods in the literature. In contrast, another study evaluated the use of ultrasonic bone curette compared with a high-speed drill in spinal surgery. Both groups experienced dural tears, and this study concluded that one method was not significantly better than the other^43^. The suggested optimal settings for vibrational drilling were noted in 1 study to be a drill speed of 1,000 rpm with a frequency of 20 kHz^75^.

### Methods of Detecting Damage When Using Orthopaedic Power Tools

To minimize iatrogenic injury, it is important to easily and rapidly identify when injury occurs intraoperatively. One novel example includes the use of a spectroscopy device integrated into a power drill to detect the bone-tissue boundary when drilling holes for intramedullary nailing^15^. This helps to reduce breaching of the periosteum and unintentional soft-tissue injury.

The use of methods providing real-time feedback is increasing. Bolger et al. used an electrical conductivity device to detect iatrogenic spinal pedicle perforation^61^. In 1 multicenter study, it demonstrated a sensitivity of >98% in the detection of breaches, 52% more when compared with the surgeon alone^62^. Similarly, a systematic review of intraoperative somatosensory-evoked potential and transcranial motor-evoked potential methods in cervical spine surgery showed a high sensitivity/specificity for both (22% to 100%/100%, and 78% to 100%/100%, respectively)^64^. Another study used stimulus-evoked electromyography to detect proximity to neural structures during iliosacral screw placement. Four of 51 screws were redirected as a consequence of the technique, and all patients had normal neurological status postoperatively^63^. Other novel methods include the use of a bioimpedance-sensing drill to successfully differentiate between cortical and cancellous bone^65^ and acoustic-emission signal analysis, which is based on the principle that different bone types will produce varying sound signals when being drilled^66^.

### Recommendations for Power-Tool Settings

With such a wide range of equipment, imaging modalities, and device settings available, there can be much heterogeneity in tools and settings used during orthopaedic procedures. Several papers have specific recommendations for power-tool equipment settings to help reduce the risk of iatrogenic injury.

With regard to drill bits, several papers agree that blunt drill bits cause higher damage to bone than sharp bits^51,52^, with 1 study demonstrating significant differences in plunging depth when sharp or blunt drill bits are used^5^. Smooth pins have also been shown to reduce the risk of overdrilling compared with threaded pins and can reduce iatrogenic injury in the form of tissue entanglement^50^.

Two studies looked at dual motor drilling. Unlike use of a conventional drill, this involves a second motor that retracts an attached sleeve at a set rate, accurately advancing the drill bit, and measures the drill bit’s energy expenditure and the distance drilled, which is continuously communicated to the surgeon. Gilmer and Lang^14^ looked at a dual motor drill for real-time measurement of torque, depth, and bone density. They found that this tool could accurately determine these parameters and, thus, give indications of screw pull-out force and cortex boundaries to prevent screw stripping and overpenetration. The second study showed a mean plunging distance of <1 mm using a dual motor drill and found that there was no difference between novice and experienced surgeons using this technique^49^. It should be noted, however, that both were preliminary studies involving artificial bone specimens and the clinical applicability of the tool would need to be validated in vivo. Other methods in the literature on detecting real-time feedback of bone conditions included the use of laser displacement sensors in a laboratory study^76^.

### Conclusions

Although iatrogenic injury in orthopaedic surgery has been described in the literature, it likely is vastly underreported. Despite this, it is important not to overlook the role of power tools in contributing to patient harm and techniques for reducing injury. Such methods should be considered in terms of equipment factors (e.g., drill speed, intraoperative imaging, use of robotic guidance), patient factors (e.g., anatomical variance, safe zones), and surgical factors (e.g., tools to increase haptic feedback, simulation training, and knowledge of critical anatomy).
